# From Evaporation to Edema: A Scoping Review of Physical and Biological Determinants of Early Fluid Distribution in Burn Patients

**DOI:** 10.3390/ebj7020021

**Published:** 2026-04-16

**Authors:** Sergio Arlati, Paolo Aseni

**Affiliations:** 1 Intensive Care Unit “G. Bozza”, ASST Grande Ospedale Metropolitano Niguarda, Piazza Ospedale Maggiore 3, 20162 Milan, Italy; sergioarlati58@gmail.com; 2 Department of Emergency Medicine, ASST Grande Ospedale Metropolitano Niguarda, Piazza Ospedale Maggiore 3, 20162 Milan, Italy

**Keywords:** burns, evaporative losses, water vapor pressure, burn dressing, environmental therapy, fluid resuscitation volume, post-burn edema

## Abstract

**Highlights:**

**What are the main findings?**

- Early post-burn evaporative losses are substantial, quantifiable, and strongly influenced by environmental conditions and wound characteristics.
- The TEWL/edema ratio shows a robust inverse relationship with resuscitation volume, identifying a shift from evaporation-dominated to edema-dominated fluid distribution.

**What are the implications of the main findings?**

- Estimating evaporative losses may improve interpretation of fluid balance and help explain discrepancies between fluid input and weight gain.
- The TEWL/edema ratio could serve as a simple physiological adjunct to guide more targeted and restrictive burn resuscitation strategies, pending prospective validation.

**Abstract:**

**Background**: Evaporative water loss from burn wounds is a major but often neglected component of early fluid requirements. Despite its physiological importance, no dedicated review has quantified acute post-burn evaporative water loss (TEWL) and its interaction with modern resuscitation strategies in over 40 years. Recent mass-casualty burn events in specialized centers have re-emphasized the clinical importance of accurate early fluid balance, which is particularly challenging. **Methods**: A scoping review (PRISMA-ScR) of historical quantitative studies and 23 contemporary (2015–2025) adult major-burn resuscitation cohorts was conducted. Expected TEWL was derived from Lamke benchmarks; interstitial edema was estimated from the only available regression of simultaneous fluid input and 24 h weight change. A novel TEWL/edema ratio was tested against resuscitation volume (mL/kg/%TBSA) and the established input/output (I/O) ratio. **Results**: In the acute phase, the median TEWL normalized to total body surface area was 71 mL/m^2^/h [52–79 mL/m^2^/h], allowing for calculation of the TEWL/edema ratio. The TEWL/edema ratio was inversely correlated with the resuscitation fluid dose (R^2^ = 0.811) and the I/O ratio as well (R^2^ = 0.86), crossing unity at 2.85 mL/kg/%TBSA. A ratio > 1 signals high evaporative drive and/or possible under-resuscitation; a ratio < 1 alerts to fluid creep before significant weight gain. **Conclusions**: The TEWL/edema ratio is the first physiology-grounded, easily calculable resuscitation endpoint that complements urine output by providing insight into whether administered fluid is lost as obligatory evaporation or sequestered as edema. Routine estimation of expected TEWL and early monitoring of the TEWL/edema ratio may help guide goal-directed burn resuscitation, especially when early excision is delayed or impossible. Given the substantial inter-individual variability, the ratio derived from aggregate data should not be interpreted as a patient-specific predictor.

## 1. Introduction

Severe burns disrupt the skin’s barrier function, leading to massive transcutaneous water and heat loss. In injuries exceeding 20–30% of total body surface area (TBSA), evaporative water loss from the wound surface becomes a relevant factor in fluid balance and a primary driver of energy expenditure [1,2]. In the first 48–72 h, total insensible losses may reach 3–4.5 L/m^2^ burned/day [3], far exceeding normal insensible losses (≈0.5–1 L/day) [4,5]. These losses, combined with capillary leak and obligatory fluid sequestration into injured tissues [6,7,8], create a narrow therapeutic window: under-resuscitation causes hypovolemia and organ failure, whereas over-resuscitation promotes profound edema, abdominal compartment syndrome, and prolonged ventilation.

Despite the critical importance of evaporative losses, their systematic quantification has largely been neglected in modern burn literature. Early excision and grafting within 48–72 h have reduced the clinical relevance of prolonged exposure in high-resource centers, while resuscitation research has focused almost exclusively on infused volumes and urinary output. Consequently, the magnitude of unmeasured evaporative losses and their interaction with resuscitation strategies remain underappreciated, especially in delayed-presentation or resource-limited settings.

Recent mass-casualty burn events in Switzerland and referral centers have highlighted this gap, with simultaneous resuscitation of multiple severely burned patients underscoring the challenges of avoiding under- and over-resuscitation without reliable evaporative loss estimates. Accurate estimation of evaporative water loss is not merely academic: it explains the frequent discrepancy between large positive fluid balance and less than expected weight gain, provides a physiological rationale for restrictive resuscitation strategies, and may serve as an indirect marker of excessive fluid administration when evaporative loss is outstripped by interstitial sequestration. Furthermore, environmental factors (temperature, humidity, and airflow) and wound coverage dramatically modulate evaporative rates [9,10,11,12], offering non-pharmacological levers to optimize fluid therapy.

This scoping review re-examines the physiology, measurement methods, and clinical implications of evaporative and transcutaneous water loss in major burns, integrating historical quantitative data with contemporary resuscitation cohorts (2015–2025). Its aims are to: (i) quantify the magnitude and time course of evaporative losses under varying conditions; (ii) clarify the influence of burn depth, dressings, grafts, and environmental therapy; and (iii) explore the relationship between administered fluid volume, evaporative loss, and edema formation as a potential guide for goal-directed burn resuscitation.

## 2. Materials and Methods

This study was conducted as a scoping review with a quantitative synthesis of selected data, following the Preferred Reporting Items for Systematic Reviews and Meta-Analyses extension for Scoping Reviews (PRISMA-ScR) guidelines [13]. The acronym “TEWL” was used operationally to denote evaporative water loss from burned or uncovered wound surfaces, rather than its classical dermatological meaning of transepidermal water loss through intact skin.

### 2.1. Search Strategy and Selection Criteria

A comprehensive literature search was performed in PubMed/MEDLINE and Embase from 1945 to 15 September 2025 with a restriction to English literature. Three separate search blocks were created:

- Evaporative and transepidermal water loss (TEWL) in burn patient MeSH/Embase terms: (“Burns” OR “Thermal Injury”) AND (“Water Loss, Insensible” OR “Evaporation” OR “Evaporative Water Loss” OR “Transepidermal Water Loss” OR “TEWL” OR “Water Vapor Pressure” OR “Insensible Loss”).
- Fluid resuscitation volumes in adult major burns (2015–2025) (“Burns” OR “Thermal Injury”) AND (“Fluid Resuscitation” OR “Parkland Formula”) AND (“1 January 2015” [Date of Publication]: “15 September 2025” [Date of Publication]).
- Post-burn body weight gain or edema quantification (“Burns” OR “Thermal Injury”) AND (“Weight Gain” OR “Burn Edema” OR “Fluid Accumulation”).

### 2.2. Eligibility Criteria

Studies were included if they provided quantitative data on evaporative/insensible water loss, total fluid volume administered in the first 24 h, or body weight change after resuscitation in humans with partial or full-thickness burns. This included:

1. Original clinical or physiological studies on adult patients (≥16 years).
2. Relevant review articles (narrative or systematic) reporting or summarizing quantitative data from original studies.
3. Technical or experimental studies on evaporative water loss (e.g., models or measurements relevant to human burns).

Pediatric-only studies were excluded due to markedly different evaporative rates per m^2^ BSA. Case reports, conference abstracts, letters, editorials, and studies without usable numerical data were excluded.

### 2.3. Study Selection

Two reviewers independently screened the titles and abstracts of all retrieved records against eligibility criteria. Full texts of potentially relevant reports were retrieved and independently assessed for eligibility by the same two reviewers. Any disagreements were resolved through discussion and consensus. No unresolved disagreement required the involvement of a third reviewer.

### 2.4. Data Extraction

Data were extracted using a pre-piloted standardized form. The following data items were collected when available: first author, year of publication, number of patients, %TBSA burned, burn depth, environmental conditions (temperature, relative humidity, airflow), method of evaporation measurement, reported evaporative/insensible water loss values, resuscitation fluid volume administered in the first 24 h (mL or mL/kg/%TBSA), and body weight change post-resuscitation.

### 2.5. Data Synthesis and Analysis

Evaporative rates originally reported in different units (g/m^2^/h, kcal/m^2^/h, mL/kg/h, etc.) were converted to mL/m^2^/h using the following constants:

- Latent heat of vaporization of water at 33–37 °C ≈ 580 kcal/L (2427 J/g).
- 1 kcal = 4.186 kJ.

When environmental parameters were provided, the skin-to-air water vapor pressure gradient was calculated using the Magnus approximation (see Supplementary File S1). The estimated TEWL for comparative purposes was derived from Lamke’s evaporative estimates [4]:

- 143 mL/m^2^ TBSA/h for III° degree burns.
- 178 mL/m^2^ TBSA/h for II° degree burns.
- Intermediate value of 160 mL/m^2^ TBSA/h when burn-depth distribution was mixed or not specified.

Edema accumulation in the first 24 h was estimated from four historical studies [14,15,16,17] reporting simultaneous fluid input and weight gain using linear regression analysis. Edema volume was estimated from weight gain after the initial 24 h of fluid resuscitation, assuming 1 kg of water equals 1 L. Linear regression was performed between measured edema volume and 24 h fluid balance (infused volume minus urinary output): edema (mL) = 0.9154 × fluid balance (mL) − 4047; R^2^ = 0.907, 9 paired observations. Because no individual-patient records were available for re-analysis, this scoping review relied exclusively on published aggregate cohort data. Consequently, revalidation of the regression equations, updating of reference values beyond the available literature, or application of multiple imputation techniques was not feasible and was not attempted. The present synthesis, therefore, relies exclusively on the transparency of the historical physiological assumptions and on the explicit acknowledgment of their limitations (see Section 4.2).

The TEWL/edema ratio was calculated for each contemporary resuscitation study to explore the relationship between evaporative loss and interstitial fluid sequestration at different resuscitation volumes. To allow calculation of a unitless TEWL/edema ratio, both parameters were normalized to body surface area and expressed as mL/m^2^ BSA/h. These normalized units were used exclusively for constructing the TEWL/edema ratio. A power-law regression was performed between 24 h resuscitation volume (mL/kg/%TBSA) and the TEWL/edema ratio. Sensitivity analyses were performed to assess the robustness of the power-law models to key parameter assumptions (Lamke rates, edema intercept, and vapor pressure gradient: see Supplementary File S2). These analyses evaluate parameter robustness only and do not substitute for prospective, individual-level validation. In prespecified sensitivity analyses, the regression was repeated after (i) restricting it to cohorts with sample sizes ≥ 30 and (ii) excluding the lowest and highest 10% of resuscitation volumes (10th–90th percentiles) to assess the influence of small cohorts and leverage points on the relationship. To further validate the physiological meaning of the TEWL/edema ratio, the hourly input/output (I/O) ratio was calculated for each cohort when cumulative 24 h fluid input and urine output were explicitly reported (18/23 studies). The I/O ratio (mL/kg/%TBSA/24 h divided by mL/kg urine produced per hour) is a well-established marker of resuscitation efficacy in burns. A formal non-linear regression was performed between the TEWL/edema ratio and the I/O ratio, expecting an inverse mathematical relationship because the two indices reflect opposite sides of the same fluid-distribution process (microvascular perfusion vs. capillary leakage).

## 3. Results

**Figure 1:**
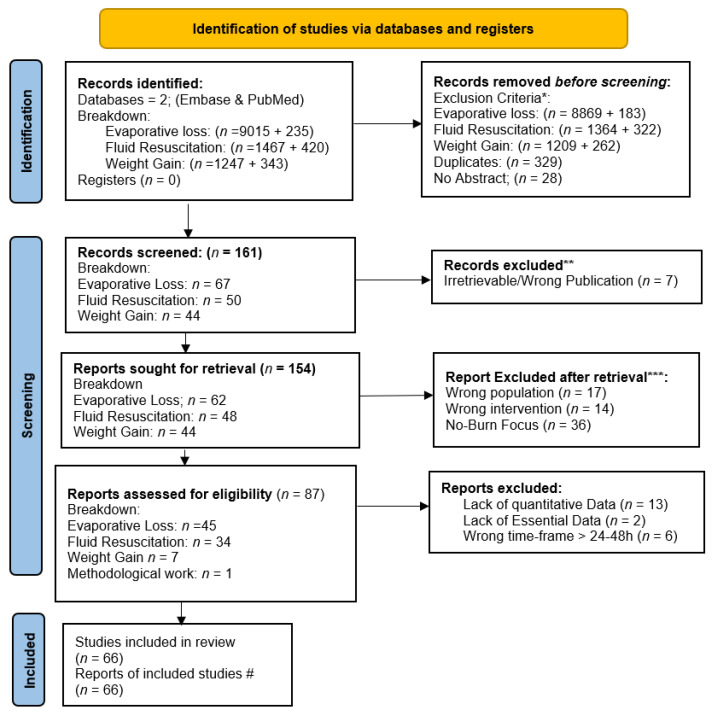
PRISMA-ScR Flow-Diagram of Study Selection for the Scoping Review on Evaporative Loss, Fluid Resuscitation and Weight Gain in burn injuries. Legend: * Exclusion criteria: non-human, non-english, pediatrics; ** automated tool; *** Full-text retrieval; # Breakdown of included studies (*n* = 66): Evaporative Losses *n* = 38 [(incl.2 book chapters, 2 technical evaporimeter, 1 animal model, 1 dressing review (cross-topic)], Fluid resuscitation *n* = 23, Weight Gain *n* = 4; Methodological work (*n* = 1). Full details in Supplementary Table S1 (Author, Year, DOI, Summary).

After screening and full-text assessment, 66 studies were included in the review across three domains: evaporative water loss (38 unique records), fluid resuscitation in burns (23 unique records), and post-burn weight gain/edema (4 unique records), methodological work (1 record). The study selection process, including screening and reasons for exclusion criteria, is summarized in the PRISMA-ScR flow diagram (Figure 1), checklist (Supplementary Table S1), and complete reference list (Supplementary Table S2).

### 3.1. Environmental and Physical Determinants of Evaporation

Evaporative water loss from burn wounds is governed by fundamental biophysical principles, representing a critical source of both fluid depletion and heat dissipation [18]. The evaporation of 1 L of water at body temperature requires approximately 580 kcal (2427 kJ), equivalent to one-third to two-thirds of the basal metabolic rate in a 70 kg adult with extensive burns. This process is driven by the water vapor pressure gradient between the wound surface (assuming saturated at skin temperature, 33–37 °C) and the surrounding air. Key determinants include:

- Ambient temperature and relative humidity (RH): Low RH and high temperatures increase the gradient, accelerating evaporation. For example, at 50% RH, air holds half its maximum moisture capacity; heating air further reduces RH, enhancing the gradient.
- Airflow: Convective currents dramatically amplify loss; airflow at 0.6 m/s (as in air-fluidized beds) can double evaporation compared to still air.
- Exposed surface area and skin wettedness: In supine patients, the effective evaporating area is approximately 0.6 × TBSA (m^2^). The wetness coefficient (ω, 0–1) reflects the fraction of the surface from which water can freely evaporate. Values ≤ 0.25 are typical of thermoneutral conditions, whereas values approaching 1.0 occur in the presence of wound exudate.

**Evaporative Water Loss (simplified equations)**

**Evaporative power** (W/m^2^): Ev = K_1_ × (P_skin_ − P_air_)

*where K*_1_ *= evaporative coefficient (airspeed-dependent); P = water vapor pressure.*

**Daily water loss** (mL/kg/day): Loss = K_2_ × Ev × BSA/weight

*where K*_2_ *= 0.035 (daily energy-to-volume conversion); BSA = body surface area.*

**Air vapor pressure** (mmHg): P_air_ = K_3_(T) × RH/100

*where K*_3_ *(T) = saturation pressure at temperature T (°C).*

These simplified equations estimate insensible losses for burn resuscitation. Full derivations and coefficients are provided in Supplementary File S1.

**Figure 2:**
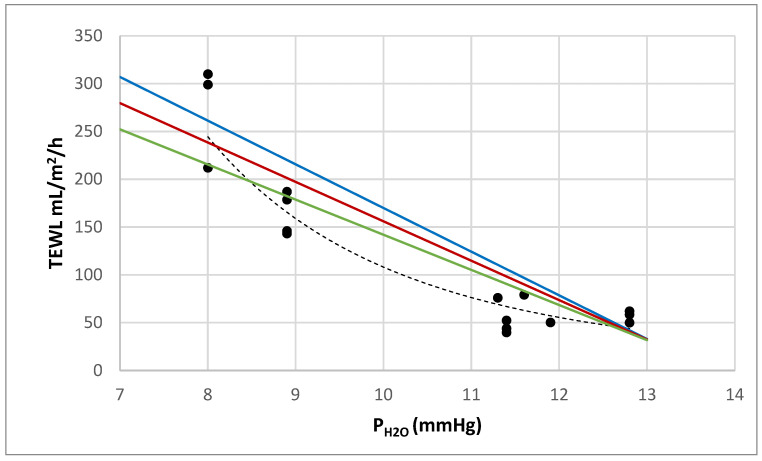
Raw scatterplot showing the relationship between ambient water vapor pressure (P_H2O_, in mmHg) and evaporative water loss rate (TEWL, in mL/m^2^/h) in burn patients, compiled from data from seven historical studies [3,4,19,20,21,22,23] (detailed in Supplementary Table S3). Points represent measurements at different post-burn times and environmental conditions. The dashed curvilinear relationship (black dotted line) does not reflect the true physiological linear relationship between P_H2O_ and TEWL. The three superimposed linear regressions—blue (t = 1), red (t = 3), and green (t = 5)—represent the results of a general linear model (GLM) that accounts for the temporal effect on the relationship between P_H2O_ and TEWL.

Analysis of seven key studies [3,4,19,20,21,22,23] (see Supplementary Table S3) under varying environmental conditions revealed an apparent non-linear relationship between ambient vapor pressure and evaporation rate in the raw scatterplot (Figure 2). An empirical power-law fit, TEWL = 1.47 × 10^6^ P_H2O_^−4.14^, achieved R^2^ = 0.916 but lacked physiological interpretability as vapor tension and evaporation are physically related by the linear “evaporative power” equation (see above). A generalized linear model incorporating a time × P_H2O_ interaction, TEWL = 656 − 47.9·P_H2O_ − 29.3 t + 2.23·(P_H2O_·t) (R^2^ = 0.911, N = 16), effectively captures this effect: the effective permeability (slope) k(t) = 47.9 − 2.23 t decreases linearly with t (days), consistent with recovery of the epidermal barrier function [24,25]. Accordingly, the TEWL–P_H2O_ regression lines show a progressive reduction in slope with increasing t, indicating a diminished TEWL response to the imposed vapor pressure gradient (Figure 2).

Low vapor pressures (drier air) markedly increase loss, confirming RH as a pivotal modulator of both fluid requirements and patient comfort. TEWL varies 2–4-fold with room T (22–30 °C), RH (20–60%), and airflow (still vs. convective) (Table 1).

The optimal ambient temperature for burn patients is approximately 32 °C [12].

### 3.2. Air-Fluidized Beds and Environmental Therapy

Air-fluidized beds, introduced in the 1970s, provide pressure relief, thermal support, and a controlled microenvironment (typically 33–36 °C, RH ≈ 40%). Warm, dry airflow creates a fluid-like interface that serves as a “drying technique,” significantly enhancing evaporation through convection. In healthy subjects, rates reach 37.7 mL/kg/h—three to four times higher than on conventional beds [26]. In burned patients, losses up to 8.1 ± 0.7 L/m^2^/day have been reported [27], exceeding classical estimates by 3.5–5 L/m^2^/day.

Recalculation under still-air conditions (airspeed 0.1 m/s, ω = 0.25) yields values aligning with traditional benchmarks [23], confirming convection as the primary amplifier [28]. Thus, while beneficial for wound healing and comfort, air-fluidized beds necessitate increased free-water supplementation and close monitoring to prevent hypernatremia and dehydration.

### 3.3. Influence of Burn Depth and Stage

Evaporative rates vary with burn depth and the healing phase. Partial-thickness burns exhibit higher initial losses (≈5500 mL/m^2^/day) due to exudation; full-thickness burns are lower (≈4000 mL/m^2^/day) owing to the insulating eschar. Rates decline over time (3000–3500 mL/m^2^/day by week 2 and 2000–2500 mL/m^2^/day by week 3 [3]), as a temporary barrier is formed [3]. Granulating wounds and donor sites show peak losses (214 ± 8.4 and 176 ± 14.5 mL/m^2^/h, respectively), falling to 60–75 mL/m^2^/h after one week with re-epithelialization [29].

### 3.4. Role of Dressings and Grafting

Wound dressing restores barrier function and modulates TEWL. Conventional gauze offers minimal insulation; occlusive dressings (e.g., hydrogels, Sulfamylon^®^ (Mylan Pharmaceuticals Inc., Canonsburg, PA, USA)) reduce loss to ≈870 mL/m^2^/day [19,30]. Biological dressings (autografts, allografts, and xenografts) achieve up to >90% reduction, mimicking intact skin (Table 2) [29]. Advanced substitutes (Matriderm^®^ (MedSkin Solutions Dr. Suwelack AG, Billerbeck, Germany), Integra^®^ (Integra LifeSciences Corporation, Princeton, NJ, USA), Biobrane^®^ (Smith & Nephew plc, London, UK)) with silicone/collagen layers yield TEWL values approaching normal skin (Table 2) [31,32,33,34]. Split-thickness skin grafts (STSGs) provide limited insulation in the early stages, especially when meshed. TEWL measurement serves as a non-invasive tool to monitor graft maturation and wound healing [35,36,37].

✓ Technical Aspects of Evaporation Measurement

Direct methods [38,39] include:

- “Evaporimetry”: Measures local vapor flux; useful for dressing evaluation but underestimates peak losses in extensive wounds and is sensitive to ambient conditions.
- “Hygrometry”: Assesses humidity above the wound; limited by air currents (open chambers) or saturation (closed systems).

Indirect estimation [3,39]:

- Utilizes high-sensitivity weighing beds via body weight change (gravimetry) to capture net water loss (including respiratory over short intervals) [40]; this is ideal for the acute phase but requires meticulous calibration. Gravimetry remains the most reliable—although cumbersome—bedside tool for total insensible losses.

### 3.5. Reliability and Clinical Implications of TEWL Estimates

**Figure 3:**
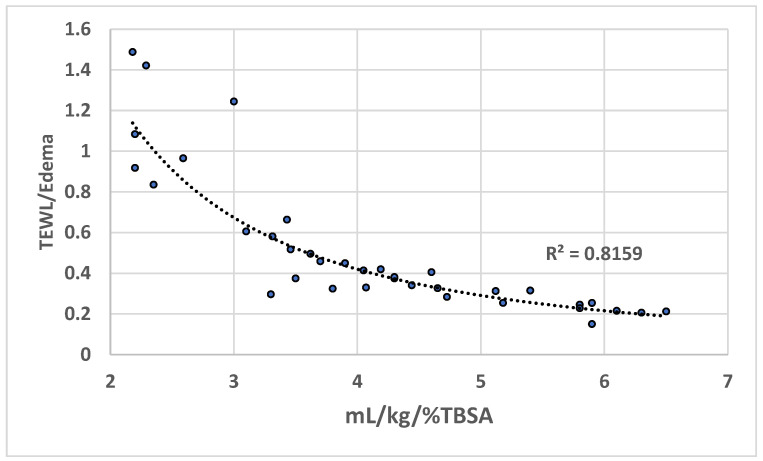
Power-law relationship between 24 h crystalloid resuscitation volume (mL/kg/%TBSA) and TEWL/edema ratio in 23 modern adult burn cohorts [41,42,43,44,45,46,47,48,49,50,51,52,53,54,55,56,57,58,59,60,61,62,63] (R^2^ = 0.816).

**Figure 4:**
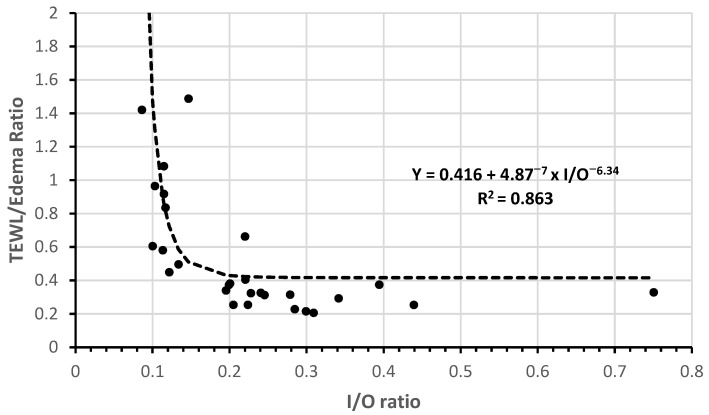
Inverse correlation between TEWL/edema ratio and hourly input/output ratio in 18 cohorts with reported urine output [41,42,43,44,45,46,47,48,49,50,51,52,53,54,55,56,57,58,59,60,61,62,63]. Unity (TEWL/edema = 1) is reached at 0.302 I/O ratio value (R^2^ = 0.863).

In 23 contemporary adult cohorts (2015–2025; median TBSA 38.6%, IQR: 32–49.5%) [41,42,43,44,45,46,47,48,49,50,51,52,53,54,55,56,57,58,59,60,61,62,63] (see Supplementary Tables S4 and S5 for detailed cohort characteristics), 24 h crystalloid administration ranged from 2.18 to 6.5 mL/kg/%TBSA (median 4.05). Expected 24 h TEWL, calculated using Lamke-derived rates adjusted for predominant burn depth (see Supplementary Table S4), had a median of 71 mL/m^2^ BSA/h (IQR: 52–79 mL/m^2^/h). Estimated interstitial edema was derived from the four historical studies that reported simultaneous fluid input, urine output and post-resuscitation weight gain (edema [mL] = 0.9154 × fluid balance [mL] − 4047; R^2^ = 0.907; see Supplementary Table S6 for details). When applied to the 23 contemporary cohorts, estimated edema yielded a median value of 165.7 mL/m^2^ BSA/h (IQR: 117–225 mL/m^2^/h). Resuscitation intensity explained 81.6% of the variance in the TEWL/edema ratio through a power-law relationship (TEWL/edema = 4.0987 × [mL/kg/%TBSA]^−1.643^; R^2^ = 0.816, *p* < 0.0001; Figure 3). More importantly, the TEWL/edema ratio showed an even stronger inverse relationship with the hourly input/output ratio in the 18 cohorts reporting urine output (R^2^ = 0.863, *p* < 0.0001; Figure 4 and Supplementary Table S5). The curves crossed unity (TEWL/edema = 1) at 2.85 mL/kg/%TBSA.

The power-law relationship remained robust across ±20–50% variations in model parameters (TEWL/edema crossing unity 1.89–4.48 mL/kg/%TBSA; R^2^ 0.70–0.88; Supplementary File S2). As analyses were conducted at the cohort level, the results cannot capture inter-individual variability in capillary leak or resuscitation response.

Similarly, the sensitivity analyses confirm the robustness of the power-law fit (TEWL/edema crossing unity 0.199–0.541 I/O ratio; R^2^ 0.721–0.921; Supplementary File S2). The sensitivity analyses performed assess parameter and model robustness within the stated assumptions and do not substitute for prospective, individual patient validation.

## 4. Discussion

This is the first dedicated review—narrative or systematic—in over 40 years to focus specifically on acute evaporative water loss in major burns and its implications for modern fluid resuscitation.

A recent mass-casualty burn event in Switzerland managed in a specialized center highlighted the clinical relevance of this issue. Interest in quantifying evaporative water loss has markedly declined over the past three decades, primarily because early tangential excision and immediate or very early grafting (within 48–72 h) have become standard in high-resource burn centers, dramatically shortening the period of exposed wound and reducing the clinical impact of prolonged evaporation. However, this paradigm does not apply universally: in low- and middle-income countries, where surgical capacity, blood-bank support, and skin-substitute availability are limited, conservative management with delayed coverage remains common, and prolonged evaporative losses increase the risk of both under-resuscitation (dehydration and hypernatremia) and fluid creep.

Even in high-income settings, a non-negligible proportion of patients with extreme TBSA (≥70–80%), inhalation injury, or delayed presentation are too unstable for early surgery; in these cases, open-wound evaporative losses regain full clinical relevance and must be actively managed to prevent dehydration, hypernatremia, and excessive resuscitation volumes. Thus, although largely solved for many, the problem of acute evaporative water loss remains highly relevant in resource-constrained environments and in the most severely injured or late-presenting patients worldwide. These losses are highly sensitive to burn depth, time since injury, wound coverage, and environmental conditions (temperature, humidity, and airflow). Occlusive dressings, biological coverings, and modern dermal substitutes can reduce TEWL by >90%, whereas convective systems such as air-fluidized beds may paradoxically increase it by a factor of 1.5–2.5 times.

This review evaluates the reliability of TEWL estimates in relation to fluid retention during the first 24 h post-injury. Both parameters increase with burn extent and originate from the same biological phenomena (enhanced permeability and fluid extravasation). However, TEWL is governed by well-defined physical laws and reflects surface-level dynamics, whereas interstitial fluid accumulation depends on volumetric factors and is driven by biological mechanisms characterized by high inter-individual variability in response. In a cylindrical structure such as the forearm, a unit increase in diameter produces a linear rise in surface area but a quadratic rise in volume. Consequently, a 1 cm increase in diameter results in an approximate 17% increase in surface area and a 36% increase in volume, highlighting the disproportionately faster growth of volume relative to surface area. A non-linear interdependence is therefore to be expected. A key observation is the inverse relationship between resuscitation volume (mL/kg/%TBSA) and the TEWL/edema ratio. At restrictive volumes (<3 mL/kg/%TBSA), evaporative loss exceeds interstitial sequestration; above the traditional Parkland target, edema formation rapidly outpaces evaporation. This shift explains the clinical paradox of marked positive fluid balance with correspondingly limited weight gain and supports the physiological rationale for restrictive or goal-directed strategies that limit interstitial overload while still covering insensible losses. These findings align seamlessly with the latest American Burn Association (ABA) Clinical Practice Guidelines on Burn Shock Resuscitation (2024) [64], which recommend initiating fluid therapy at 2 mL/kg/%TBSA to minimize total volumes and edema-related complications, with albumin reserved for rescue scenarios when crystalloids fail [64]. The guidelines highlight the paucity of evidence for adjuncts like high-dose vitamin C or vasopressors, underscoring the need for simple, physiology-based endpoints to guide titration—a gap our TEWL/edema ratio addresses directly. A recent state-of-the-science analysis notes that while monitoring innovations (e.g., lactate and computerized supports) have mixed results in curbing fluid creep, no tool yet quantifies unmeasured evaporative losses as a counterbalance to sequestration [65]. Similarly, a 2025 review comparing burns and sepsis resuscitation identifies persistent uncertainty in fluid endpoints, with burns lagging behind sepsis in adopting goal-directed strategies [66]. The TEWL/edema ratio shows a strong inverse correlation with both resuscitation volume and the established I/O ratio. However, correlation does not imply causation, and the ratio should therefore be regarded as a hypothesis-generating physiological indicator, rather than a clinically validated predictive tool. Likewise, the regression-based edema estimate represents a heuristic-physiologically grounded approximation rather than a validated bedside predictor. Therefore, this scoping review cannot provide direct evidence of clinical efficacy or outcome benefit and should be interpreted accordingly. Prospective cohort studies with individual-patient data will be essential to confirm its clinical efficacy and to evaluate the performance in clinically relevant subgroups (e.g., burns ≥ 70% TBSA, delayed presentation, concomitant inhalation injury, rescue colloid administration, and pediatric patients). Such analyses will help determine whether the ratio retains its physiological interpretability and potential utility across these heterogeneous clinical scenarios.

The prespecified sensitivity analyses show that the inverse relationship between the resuscitation volume and the TEWL/edema ratio is not driven by a few small or extreme cohorts; both the slope and the crossing point around 3 mL/kg/%TBSA remain essentially unchanged, reinforcing the physiological interpretation of an evaporation-to-edema shift beyond restrictive dosing. We performed sensitivity analyses to evaluate the robustness of the power law to key parameter assumptions (Lamke rates, edema intercept, and vapor pressure gradient); these analyses test parameter sensitivity only, cannot account for unmeasured clinical confounders, and do not replace prospective, individual-level validation.

Taken together with the independent correlation against the I/O ratio (R^2^ = 0.863), this robustness supports using the TEWL/edema ratio as a heuristic distribution endpoint rather than a fragile artifact of particular datasets. Prospective validation will determine whether this evaporative-adjusted metric can assist the operationalization of restrictive resuscitation strategies and retain its physiological interpretability across clinically relevant subgroups.

The TEWL/edema ratio and the classic hourly input/output (I/O) ratio represent two sides of the same physiological coin: the former quantifies the proportion of administered fluid that is lost as obligatory evaporation versus sequestered as interstitial edema, whereas the latter reflects end-organ perfusion through the urinary response. Across the 18 cohorts reporting cumulative 24 h urine output, the relationship between the two indices was remarkably tight and followed the predicted inverse non-linear pattern (TEWL/edema = 0.416 + 4.87 × 10^−7^ × (I/O ratio)^−6.34^; R^2^ = 0.863, *p* < 0.0001; Figure 4; Table 3).

When TEWL/edema exceeded 1.0 (evaporation-driven regimen), the I/O ratio was consistently below 0.302 (≈3.2 mL/kg/%TBSA); conversely, when the TEWL/edema ratio fell below 1 (edema-driven regimen), the I/O ratio rose above 0.302. This strong correlation observed in completely independent datasets supports the idea that the TEWL/edema ratio may capture underlying physiological patterns of fluid distribution, indicating a potential role as an early marker of fluid creep. If prospectively validated, such an index could help clinicians detect possible over-resuscitation as early as 8 h and guide titration toward more restrictive targets.

### 4.1. Clinical Impact

The potential clinical value of the TEWL/edema ratio lies in its ability to serve as a simple, zero-cost physiological index that may help clinicians interpret fluid distribution during the first 24 h after injury. If prospectively validated, it could complement urine output by indicating whether administered fluid is predominantly lost through evaporation or sequestered as interstitial edema.

In practical terms, it could help answer the crucial clinical question every burn clinician asks during the first 24 h: “Where is the fluid I am giving actually going?”

- Ratio ≈ 1 (achieved at ≈2.85 mL/kg/%TBSA in the cohorts): The infused volume is physiologically appropriate; it replaces evaporative + urinary + baseline losses with obligate interstitial sequestration.
- Ratio > 1 (achieved at <2.85 mL/kg/%TBSA): Evaporative losses exceed the estimated fluid volume accumulation. Although effective, it might reflect inadequate resuscitation volume (verify the I/O ratio); appropriate measures should include increasing fluid rate, raising ambient humidity, applying temporary occlusive dressings, or adding free-water supplementation (especially on air-fluidized beds).
- Ratio < 1 (typically when >4 mL/kg/%TBSA): Most of the additional fluid is being sequestered as edema; immediate actions include restriction of crystalloid infusion and/or consideration of early colloid rescue or albumin, switching to a permissive oliguria strategy, or preparing for abdominal decompression if the ratio continues to fall.

**Figure 5:**
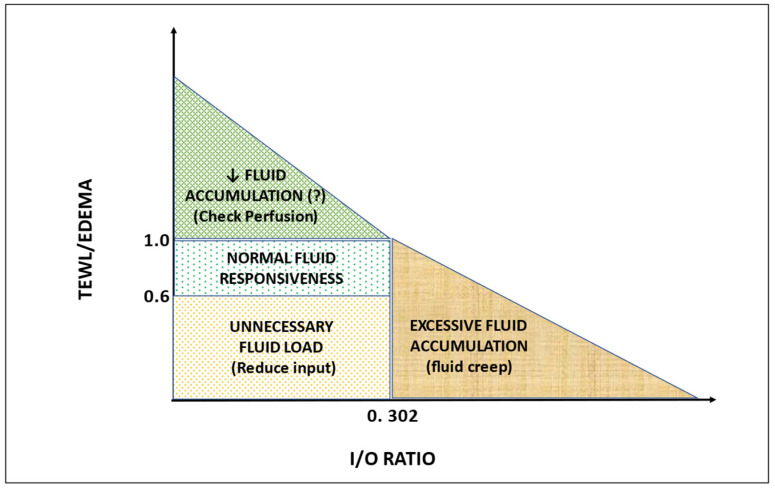
Fluid resuscitation scenarios—I/O ratio vs. TEWL/edema distribution. Legend: I/O ratio determines resuscitation efficacy; TEWL/edema ratio determines extravascular distribution (evaporation vs. sequestration).

A synoptic representation of possible scenarios and their corresponding underlying mechanisms is shown in Figure 5.

Because expected TEWL can be calculated in under a minute from %TBSA, burn depth, temperature and humidity, the TEWL/edema ratio could, in principle, be projected as early as 6–8 h and updated hourly. The worksheet provided in Supplementary File S3 is intended to facilitate prospective research and hypothesis testing; it is not a validated clinical protocol and should not be used for bedside decision-making without prospective, individual-patient validation. When considered alongside urine output and lactate clearance, TEWL may offer an additional physiological perspective on fluid distribution, potentially helping distinguish oliguria due to hypovolemia from early fluid creep. In centers with routine early excision, the ratio might help limit unnecessary fluid loading in the short pre-operative window. In low-resource settings or in ultra-major burns (>70% TBSA) where coverage is delayed, systematic estimation of expected TEWL and monitoring of the ratio could provide a pragmatic, zero-cost aid to clinical reasoning, helping avoid both dehydration/hypernatremia and excessive interstitial edema.

A paradox of the available evidence is that the two populations in which an evaporative-adjusted index might be most informative—pediatric patients and those treated in low-resource settings—are not represented in current datasets. Pediatric cohorts were intentionally excluded because evaporative rates and fluid distribution physiology differ substantially from adults, while contemporary quantitative TEWL measurements from low-resource settings are virtually absent. As a result, the applicability of the TEWL/edema ratio in these groups remains uncertain and will require dedicated prospective evaluation. Nevertheless, the biophysical principles governing evaporative water loss are universal, suggesting that the conceptual framework may retain relevance across diverse clinical environments.

### 4.2. Limitations

This scoping review is limited by its reliance on historical gravimetric and evaporimetric data and by the absence of individual-patient records. The edema regression model is based on nine cohort-level means from four pre-2011 studies that have never been replicated, creating a risk of overfitting and limiting external validation. Similarly, TEWL benchmarks derive largely from Lamke and Davies, whose measurements were performed under warm, dry conditions that maximize total non-renal water loss; although methodologically historical, these values remain the only whole-body estimates available for early resuscitation. All analyses were conducted at the cohort level, precluding assessment of inter-individual variability in capillary permeability, inhalation injury, vasoactive support, albumin timing, or environmental conditions and introducing potential ecological bias. Formal subgroup analyses were not feasible, and mathematical coupling between fluid input, weight gain, and the derived ratio cannot be fully eliminated. Generalizability is further restricted by the deliberate exclusion of pediatric patients and by the paucity of quantitative data from low- and middle-income countries. Dedicated prospective studies with direct bedside weight monitoring, updated environmental measurements, and contemporary wound-management strategies will be essential to revalidate and refine these models using individual-patient data.

## 5. Conclusions and Future Directions

Despite relying on historical gravimetric and evaporimetric techniques, these remain the only methods capable of capturing total insensible losses at a whole-body level. Quantifying evaporative water loss offers a physiologically grounded complement to current resuscitation practices, particularly in settings where early coverage is not feasible or where avoidance of fluid creep is a priority. Future prospective studies incorporating direct gravimetric measurements, updated environmental conditions, and contemporary wound-management strategies will be essential to refine TEWL reference values, revalidate the edema model, and assess the clinical utility of the TEWL/edema ratio across well-defined adult and pediatric populations.

## Figures and Tables

**Table 1:** Predicted TEWL across temperature, humidity, and airflow conditions.

T (°C)	RH 20% (Still)	RH 20% (Conv)	RH 60% (Still)	RH 60% (Conv)
22	152	274	118	213
26	176	317	137	247
30	205	369	160	288

Legend: predicted transepidermal water loss (TEWL mL/m^2^/h) from burned surfaces (wound T = 33 °C). Derived from Lamke rates (143–178 mL/m^2^/h), scaled for vapor pressure deficit (Magnus equation) and airflow (still air: h_2_ 2.2 g/s·m^−2^·°C^−1^, convective ≈ 4.0 g/s·m^2^·°C^−1^ at 0.6 m/s). Example: 22 °C/RH 20% increase from 152 mL/m^2^/h (still) to 274 mL/m^2^/h (convective).

**Table 2:** Evaporative water loss (TEWL, mean ± SD, mL/m^2^/h) with various biological, biosynthetic, and synthetic wound dressings applied to burn wounds, donor sites, and granulating wounds, compared to open burn sites. Values in parentheses indicate percentage reduction relative to uncovered burn wounds at the corresponding site (adapted/complied from references [29,31,32,33,34,35,36,37]).

	BURN SITE	DONOR SITE	GRANULATING SITE
BIOLOGICAL			
Homograft	24 ± 6 (−91%)	35 ± 6 (−87.7%)	36 ± 6 (−86.6%)
Xenograft	213 ± 30 (−15.1%)	224 ± 18 (−22.5%)	242 ± 23 (−12.9%)
Mesh	35 ± 8 (−83.5%)	30 ± 4 (−87.8%)	40 ± 6 (−86.5%)
Fetal	165 ± 13 (−15.3%)	199 ± 24 (−19.1%)	222 ± 13 (−14.6%)
BIOSYNTHETIC	No STSG	With STSG	
Biobrane^®^	110–165	−40/60%	
Matriderm^®^		12.5 ± 5.1	
Megaderm^®^		10.9 ± 6.4	
Integra^®^		<15	
SYNTHETIC			
Omiderm^®^		20	
Novosorb BTM^®^		<15	

Megaderm^®^—L&C BIO Co., Ltd., Seongnam, Republic of Korea; Omiderm^®^—Mölnlycke Health Care AB, Gothenburg, Sweden; NovoSorb BTM^®^—PolyNovo Limited, Port Melbourne, Australia.

**Table 3:** Predicted TEWL: edema ratios across fluid resuscitation doses (mL/kg/%TBSA).

TEWL/Edema	mL/kg/%TBSA	TEWL:Edema
1	2.85	1:1
0.8	3.56	1:1.25
0.6	4.3	1:1.66
0.5	5.7	1:2.00
0.45	6.3	1:2.22
0.4	≥7.0	1:2.5

Legend: TEWL/edema = 1:1 indicates that 1 mL of edema accumulates for every mL of TEWL. Example: when TELW/edema = 0.4, every liter of TEWL entails 2.5 L of sequestered edema.

## Data Availability

No new data were created or analyzed in this study. Data sharing is not applicable to this article.

## Supplementary Materials

The following supporting information can be downloaded at https://www.mdpi.com/article/10.3390/ebj7020021/s1, The Supplementary Materials contain additional tables and methodological details; all references cited in the Supplementary Materials are included in the main reference list. Table S1: PRISMA-ScR (Preferred Reporting Items for Systematic reviews and Meta-Analyses extension for Scoping Reviews) checklist; Table S2: Complete list of all 64 references; Table S3: Summary of data reported in seven relevant articles focusing on evaporative and heat losses during the early phase of burn injury; Table S4: Cohort characteristics, resuscitation volumes and Lamke-derived transepidermal water loss in contemporary adult burn series (2015–2025); Table S5: Detailed 24 h input–output balance, estimated interstitial edema and TEWL/edema ratios in contemporary adult burn resuscitation cohorts; Table S6: Historical datasets underpinning the edema regression model (fluid input, urine output, and weight gain) and derivation of the interstitial edema equation; File S1: Evaporative flux and daily water loss: fundamental equations; File S2: Sensitivity analysis; File S3: Bedside worksheet.
