# Supplementary material for: From Evaporation to Edema: A Scoping Review of Physical and Biological Determinants of Early Fluid Distribution in Burn Patients
Source: Eur Burn J. 2026 Apr 16;7(2):21. doi: 10.3390/ebj7020021 (PMC13108070; doi:10.3390/ebj7020021)
Supplement: Supplementary file 1 [file ebj-07-00021-s001.zip › ebj-4169852-supplementary.pdf]

Supplementary Table S1: PRISMA-ScR (Preferred Reporting Items for Systematic reviews and Meta-Analyses extension for Scoping Reviews) Checklist

SECTION	ITEM	PRISMA-ScR CHECKLIST ITEM	REPORTED ON PAGE #
**TITLE**			
Title	1	Identify the report as a scoping review.	Page 1 (Title)
**ABSTRACT**			
Structured summary	2	Provide a structured summary that includes (as applicable): background, objectives, eligibility criteria, sources of evidence, charting methods, results, and conclusions that relate to the review questions and objectives.	Page 1 (Abstract)
**INTRODUCTION**			
Rationale	3	Describe the rationale for the review in the context of what is already known. Explain why the review questions/objectives lend themselves to a scoping review approach.	Pages - 2
Objectives	4	Provide an explicit statement of the questions and objectives being addressed with reference to their key elements (e.g., population or participants, concepts, and context) or other relevant key elements used to conceptualize the review questions and/or objectives.	Page 2
**METHODS**			
Protocol and registration	5	Indicate whether a review protocol exists; state if and where it can be accessed (e.g., a Web address); and if available, provide registration information, including the registration number.	Not Applicable
Eligibility criteria	6	Specify characteristics of the sources of evidence used as eligibility criteria (e.g., years considered, language, and publication status), and provide a rationale.	Pages 2-3
Information sources*	7	Describe all information sources in the search (e.g., databases with dates of coverage and contact with authors to identify additional sources), as well as the date the most recent search was executed.	Page 3
Search	8	Present the full electronic search strategy for at least 1 database, including any limits used, such that it could be repeated.	Page 3
Selection of sources of evidence†	9	State the process for selecting sources of evidence (i.e., screening and eligibility) included in the scoping review.	Page 3
Data charting process‡	10	Describe the methods of charting data from the included sources of evidence (e.g., calibrated forms or forms that have been tested by the team before their use, and whether data charting was done independently or in duplicate) and any processes for obtaining and confirming data from investigators.	Page 3
Data items	11	List and define all variables for which data were sought and any assumptions and simplifications made.	Page3
Critical appraisal of individual sources of evidence§	12	If done, provide a rationale for conducting a critical appraisal of included sources of evidence; describe the methods used and how this information was used in any data synthesis (if appropriate).	Not Applicable
Synthesis of results	13	Describe the methods of handling and summarizing the data that were charted.	Pages 3-4
**RESULTS**			
Selection of sources of evidence	14	Give numbers of sources of evidence screened, assessed for eligibility, and included in the review, with reasons for exclusions at each stage, ideally using a flow diagram.	Page 4

SECTION	ITEM	PRISMA-ScR CHECKLIST ITEM	REPORTED ON PAGE #
Characteristics of sources of evidence	15	For each source of evidence, present characteristics for which data were charted and provide the citations.	Pages 4-5
Critical appraisal within sources of evidence	16	If done, present data on critical appraisal of included sources of evidence (see item 12).	Not Applicable
Results of individual sources of evidence	17	For each included source of evidence, present the relevant data that were charted that relate to the review questions and objectives.	Pages 5-8
Synthesis of results	18	Summarize and/or present the charting results as they relate to the review questions and objectives.	Pages 6-9
**DISCUSSION**			
Summary of evidence	19	Summarize the main results (including an overview of concepts, themes, and types of evidence available), link to the review questions and objectives, and consider the relevance to key groups.	Pages 9-12
Limitations	20	Discuss the limitations of the scoping review process.	Page 13
Conclusions	21	Provide a general interpretation of the results with respect to the review questions and objectives, as well as potential implications and/or next steps.	Page 13
**FUNDING**			
Funding	22	Describe sources of funding for the included sources of evidence, as well as sources of funding for the scoping review. Describe the role of the funders of the scoping review.	Not Applicable

JBI = Joanna Briggs Institute; PRISMA-ScR = Preferred Reporting Items for Systematic reviews and Meta-Analyses extension for Scoping Reviews.

* Where *sources of evidence* (see second footnote) are compiled from, such as bibliographic databases, social media platforms, and Web sites.

† A more inclusive/heterogeneous term used to account for the different types of evidence or data sources (e.g., quantitative and/or qualitative research, expert opinion, and policy documents) that may be eligible in a scoping review as opposed to only studies. This is not to be confused with *information sources* (see first footnote).

‡ The frameworks by Arksey and O'Malley (6) and Levac and colleagues (7) and the JBI guidance (4, 5) refer to the process of data extraction in a scoping review as data charting.

§ The process of systematically examining research evidence to assess its validity, results, and relevance before using it to inform a decision. This term is used for items 12 and 19 instead of "risk of bias" (which is more applicable to systematic reviews of interventions) to include and acknowledge the various sources of evidence that may be used in a scoping review (e.g., quantitative and/or qualitative research, expert opinion, and policy document).

From: Tricco AC, Lillie E, Zarin W, O'Brien KK, Colquhoun H, Levac D, et al. PRISMA Extension for Scoping Reviews (PRISMAScR): Checklist and Explanation. Ann Intern Med. 2018;169:467–473. doi: 10.7326/M18-0850.

Supplementary Table S2 – Complete List of All 66 References

#	First Author	Year	Journal	DOI or PMID	Type/Role in the Review
1	Harrison HN	1964	Surgery	PMID:14174738	Historical TEWL & energy metabolism
2	Hussain A	2013	Burns	doi:10.1016/j.burns.2013.04.026	Prognostic factors in thermal burns
3	Davies JWL	1974	Br J Plast Surg	doi:10.1016/0007-1226(74)90031-9	Guide to non-renal water loss
4	Lamke LO	1977	Burns	doi:10.1016/0305-4179(77)90004-3	TEWL from burns & grafts
5	Lamke LO	1971	Acta Derm Venereol	doi:10.2340/0001555551111119	Water evaporation from normal skin
6	Wurzer C	2018	Total Burn Care (book chapter)	ISBN:9780323476614	Pathophysiology of burn shock and edema
7	Turk E	2014	J Burn Care Res	doi:10.1097/BCR.0b013c318290124a.	Flow-mediated dilation in burn injury
8	Zetterstrom H	1980	Acta Anaesthesiol Scand	doi:10.1111/j.1399-6576.1980.tb01550.x	Plasma oncotic pressure post-burn
9	Salisbury RE	1980	Ann Plast Surg	doi:10.1097/00000637-198010000-00004	Biological dressings & TEWL
10	Martin CJ	1985	Clin Phys Physiol Meas	doi:10.1088/0143-0815/6/4/005	Computer simulation of thermal environment
11	Wilson JS	1965	Ann Surg	doi:10.1097/00000658-196507000-00019	Vapor pressure of burned skin
12	Lamke LO	1971	| Scand J Plast Reconstr Surg	doi:10.3109/02844317109042942	Evaporative loss under different conditions
13	Tricco AC	2018	Ann Intern Med	doi:10.7326/M18-0850	PRISMA-ScR guideline for scoping review
14	Busche MN	2016	Ann Plast Surg	doi:10.1097/SAP.0000000000000845	Evaporative loss in superficial/full-thickness burns
15	Du Ge-Bing	1991	Burns	doi:10.1016/0305-4179(91)90035-G	Weight gain & resuscitation regimens
16	O'Mara MS	2005	J Trauma	doi:10.1097/01.TA.0000169917.58466.24	Crystalloids vs colloids & pressures
17	Tanaka H	2000	Arch Surg	doi:10.1001/archsurg.135.3.326	Ascorbic acid & volume reduction
18	Vlachau E	2010	Burns	doi:10.1016/j.burns.2009.08.012	Hydroxyethyl starch supplementation
19	Moncrief JA	1964	Surg Forum	doi:10.197/00005373-196403000-00005	Evaporative water loss
20	Caldwell FT	1981	Ann Surg	doi:10.1097/00000658-198105000-00007	Occlusive dressings in burned children
21	Zawacki BE	1970	Ann Surg	doi:10.197/00000658-197002000-00011	Increased evaporation & hypermetabolism
22	Fallon RH	1963	Ann Surg	doi:10.197/00000658-196312000-00001	Insensible perspiration through burned skin
23	Wilmore DH	1975	J Appl Physiol	doi:10.1152/jappl.1975.38.4.593	Ambient temperature & heat production
24	Matoltsy AG	1962	J Invest Dermatol	doi:10.1038/jid.1962.45	Skin barrier regeneration
25	Carnes RW	1981	J Burn Care Rehabil	doi:10.1097/00004630-198109000-00002	Evaporative loss from healed burns
26	Micheels J	1983	Burns	doi:10.1016/0305-4179(83)90032-3	Water & sodium balance on air-fluidized bed
27	Micheels J	1983	Burns	doi:10.1016/0305-4179(83)90075-x	Air-fluidized bed physiology
28	Wenger CB	1996	Handbook of Physiology (book chapter)	ISBN-10:0195074920	Body temperature regulation
29	Lamke LO	1971	Scand J Plast Reconstr Surg	doi:10.3109/02844317109042943	Skin grafts & evaporation
30	Zawacki BE	1969	Ann Surg	doi:10.1097/00000658-196902000-00011	Sulfamylon & insensible loss

31	*Lee MC*	*2017*	*J Hand Surg Am*	*doi:10.1016/j.jhsa.2017.01.026*	*Acellular dermal matrices & TEWL*
32	*Min JH*	*2014*	*Arch Plast Surg*	*doi:10.5999/aps.2014.41.04.330*	*Matriderm & skin grafts*
33	*Greenwood JE*	*2012*	*J Burn Care Res*	*doi:10.1097/BCR.0b013e318233fac1*	*Biodegradable matrix vs Integra*
34	*Behar D*	*1986*	*J Biomed Mater Res*	*doi:10.1002/jbm.820200607*	*Omiderm wound covering*
35	*Gardien KL*	*2016*	*Burns*	*doi:10.1016/j.burns.2016.04.018*	*TEWL in burn scars*
36	*Chattopadhyay D*	*2024*	*Sci Rep*	*doi:10.1038/s41598-024-74426-0*	*TEWL & wound closure*
37	*Danielsen PL*	*2013*	*Acta Derm Venereol*	*doi:10.2340/00015555-1455*	*Erythema in skin graft donor sites*
38	*Klotz T*	*2022*	*Skin Res Technol*	*doi:10.1111/srt.13159.Epub2022.Apr12*	*Devices for TEWL measurement*
39	*Ferguson JC*	*1991*	*Clin Phys Physiol Meas*	*doi:10.1088/0143-0815/12/2/003*	*Burn wound evaporation methods*
40	*Davies JWL*	*1982*	*Physiological Responses to Burn Injury (book)*	*ISBN 10:0122060806*	*Physiological responses overview*
41	*Belba MK*	*2021*	*Burns*	*doi:10.1016/j.burns.2021.07.003*	*Modern Resuscitation*
42	*Meuli JF*	*2022*	*J Burn Care Res*	*doi:10.1093/jbcr/irab072*	*Modern Resuscitation*
43	*Le Tacon S*	*2023*	*Burns*	*doi:10.1016/j.burns.2023.02.001*	*Hematocrit as marker of fluid management*
44	*Greenhalgh DG*	*2023*	*Am Surg*	*doi:10.1097/SLA.0000000000005166*	*ABRUPT Multicenter Trial*
45	*Chiao H-Y*	*2018*	*Ann Plast Surg*	*doi:10.1097/SAP.0000000000001288*	*Goal-directed resuscitation*
46	*Tridente A*	*2025*	*Eur Burn J*	*doi:10.3390/ebj6030040*	*Fluid resuscitation practices UK*
47	*Lindhal L*	*2023*	*Burns Open*	*doi:10.1016/j.burns.2023.03.006*	*Parkland leads to high volumes*
48	*Gardiner B*	*2025*	*Burns*	*doi:10.1016/j.burns.2025.107610*	*Lower volumes & outcomes*
49	*Abdelmootal AM*	*2024*	*Burns*	*doi:10.1016/j.burns.2023.06.015*	*HES vs albumin in burns*
50	*Kahn SA*	*2025*	*J Am Coll Surg*	*doi:10.1097/XCS.0000000000001339*	*Fluid restriction & plasma*
51	*Adibfar A*	*2021*	*Burns*	*doi:10.1016/j.burns.2020.09.005*	*Vasopressors in burns*
52	*Aigner A*	*2025*	*Burns*	*doi:10.1016/j.burns.2025.107397*	*Parkland retrospective analysis*
53	*Comish P*	*2021*	*Burns*	*doi:10.1016/j.burns.2021.05.013*	*Colloid rescue & perfusion*
54	*Xiao S*	*2024*	*Eur J Med Res*	*doi:10.1186/s40001-024-01859-0*	*Inhalation injury & fluid*
55	*Zhu Y*	*2021*	*Ann Palliat Med*	*doi:10.21037/apm-21-2535*	*Pulse contour & prognosis*
56	*Tan J*	*2021*	*J Burn Care Res*	*doi:10.1093/jbcr/iraa207*	*New resuscitation formula*
57	*Boehm D*	*2019*	*J Burn Care Res*	*doi:10.1093/jbcr/irz058*	*Fluid & abdominal compartment syndrome*
58	*Lin J*	*2018*	*J Burn Care Res*	*doi:10.1093/jbcr/iry003*	*Ascorbic acid in burn shock*
59	*Lindsey L*	*2020*	*Ann Burns Fire Disasters*	*PMID:33110723*	*Adjusted body weight & FFP rescue*
60	*Liu NT*	*2018*	*J Burn Care Res*	*doi: 10.1093/jbcr/iry021*	*Predicting burn wound healing*
61	*Liu NT*	*2016*	*J Trauma Acute Care Surg*	doi: 10.1097/TA.0000000000001166.	*Predicting full-thickness proportion*
62	*Rosenthal*	*2018*	*J Burn Care Res*	*doi: 10.1016/j.burn.2018.06.002*	*Effect of obesity on burn resuscitation*
63	*Kruse*	*2025*	*Eur Burn J*	doi:10.3390/ebj6020035	*Fluid administration retrospective analysis*
64	*Cartotto R*	*2024*	*J Burn Care Res*	*doi:10.1093/jbcr/irad125*	*ABA 2024 Clinical Practice Guidelines on Burn Shock*
65	*Cartotto R*	*2022*	*J Burn Care Res*	*doi:10.1093/jbcr/irac025*	*State-of-the-art review on burn shock resuscitation*
66	*Zhou D*	*2025*	*Front Med (Lausanne)*	*doi:10.3389/fmed.2025.1561619*	*Bibliometric analysis of burns vs sepsis resuscitation*

Supplementary Table S3: Summary of data reported in seven relevant articles focusing on evaporative and heat losses during the early phase of burn injury.

Author (Year)	*Heat loss Kcal/m²TBSA/h*	*Evap Loss ml/m²TBSA/h*	*Temp (°C)*	*Rel Hum (%)*	*P_{H2O} mmHg*	*Method (meas.)*	*Time*	*Notes*
Davies (1974)	*108.7*	*187*	*32*	*25*	*8.9*	*BWL*	*1st Week*	*II° degree*
	84.5	*146*	*32*	*25*	*8.9*	*BWL*	*1st Week*	*III° degree*
Lamke (1977)	*103.2*	*178.1*	*32*	*25*	*8.9*	*EVAP*	*1st Week*	*II° degree*
	82.9	*143.2*	*32*	*25*	*8.9*	*EVAP*	*1st Week*	*III° degree*
Busche (2016)	*36.3*	*62.0*	*30*	*40*	*12.8*	*EVAP*	*24 h*	*II° (superf)*
	34.2	*58.5*	*30*	*40*	*12.8*	*EVAP*	*24 h*	*II° (deep)*
	29.5	*50.0*	*30*	*40*	*12.8*	*EVAP*	*24 h*	*III° degree*
Caldwell (1981)	*44.5*	*76*	*28*	*40*	*11.3*	*BWL*	*NR*	*-*
Zawacki (1970)	*30.0*	*52.3*	*26*	*45*	*11.4*	*BWL*	*1st Week*	*-*
	23.0	*39.8*	*26*	*45*	*11.4*	*BWL*	*2nd Week*	*-*
	25.5	*44.0*	*26*	*45*	*11.4*	*BWL*	*3rd Week*	*-*
Fallon (1963)	*180*	*310*	*22.5*	*Normal*	*8.0*	*KAL*	*6 h*	*II° degree*
	123	*212*	*22.5*	*Normal*	*8.0*	*KAL*	*12 h*	*III° degree*
	174	*299*	*22.5*	*Normal*	*8.0*	*KAL*	*6-12 h*	*Donor site*
Wilmore (1975)	*29.2*	*50.3*	*25*	*50*	*11.9*	*BWL*	*Day 9-10*	*-*
	45.9	*79.1*	*33*	*31*	*11.6*	*BWL*	*Day 9-10*	*-*

Legend: BWL body weight loss, EVAP evaporation, KAL Calorimetry, Normal (assumed 40%), (Magnus Equation): P_{H2O} (mmHg) = [0.61094·exp(17.625·T(°C)/T(°C)+243.04)]·RH·7.5

Supplementary Table S4 – Cohort characteristics, resuscitation volumes and Lamke-derived trans-epidermal water loss in contemporary adult burn series (2015–2025).

Author<br>(Year)	*N*	*Total<br>TBSA<br>(%)*	*II°<br>Burns<br>(%)*	*III°<br>Burns<br>(%)*	*ml/kg/<br>%TBSA*	*Lamke Base<br>Rate<br>ml/m²TBSA/h*	*Base Rate<br>(Weighted<br>Calculations)*	*Hourly<br>Base Rate<br>ml/m²TBSA*	*Final<br>TEWL<br>(L/day)<br>**(#)***	*Actual<br>TEWL<br>ml/h*
Belba<br>(2021)	*22*	*39*	*NR*	*NR*	*3.10*	*160<br>intermediate*	*NA*	*160*	*2758*	*115*
Meuli<br>(2021)	*29<br>10*	*60<br>84*	*30<br>25.4*	*30<br>58.6*	*5.8<br>5.9*	*178 (II°)<br>143 (III°)*	*(178x30 + 143x30) /60<br>(178x25.4 + 143x58.6) /84*	*160.5<br>153.6*	*4423<br>4714*	*184<br>196*
Le Tacon<br>(2023)	*230*	*38.6*	*16.2*	*22.4*	*4.3*	*178 (II°)<br>143 (III°)*	*(178x16.2 + 143x22.4)<br>/38.5*	*157.7*	*2666*	*111*
Greenhalgh<br>(2023)	*579*	*31.6*	*23.6*	*8*	*4.6*	*178 (II°)<br>143 (III°)*	*(178x23.6 + 143x8) /31.6*	*169.1*	*2693*	*112*
Chiao<br>(2018)	*13*	*68*	*59*	*9*	*3.6*	*178 (II°)<br>143(III°)*	*(178x59 + 143x9) /68*	*173.3*	*4810*	*200*
Tridente<br>(2025)	*198*	*27.5*	*NR*	*NR*	*3.4*	*160<br>intermediate*	*NA*	*160*	*2070*	*86*
Lindhal<br>(2023)	*46*	*32*	*NR*	*NR*	*5.9*	*160<br>intermediate*	*NA*	*160*	*2408*	*100*
Gardiner<br>(2025)	*30<br>14<br>80*	*32<br>42<br>33*	*NR<br>NR<br>NR*	*NR<br>NR<br>NR*	*2.2<br>3.5<br>5.9*	*160<br>Intermediate*	*NA<br>NA<br>NA*	*160<br>160<br>160*	*2482<br>3274<br>2522*	*103<br>136<br>105*
Abdelmootal<br>(2024)	*26<br>26*	*34.1<br>33.5*	*NR<br>NR*	*NR<br>NR*	*6.3<br>6.1*	*160<br>Intermediate*	*NA<br>NA*	*160<br>160*	*2687<br>2560*	*112<br>107*
Kahn<br>(2025)	*213*	*31.8*	*NR*	*NR*	*3.3*	*160<br>intermediate*	*NA*	*160*	*2302*	*96*
Adibifar<br>(2021)	*2822*	*30.8*	*10.4*	*20.4*	*5.1*	*178 (II°)<br>143 (III°)*	*(178x10.4 + 143x20.4)<br>/30.8*	*154.8*	*2246*	*93*
Aigner<br>(2025)	*892<br>444*	*23<br>40*	*NR<br>NR*	*NR<br>NR*	*3.0<br>3.7*	*160<br>Intermediate*	*NA<br>NA*	*160<br>160*	*1740<br>3026*	*72<br>126*
Comish<br>(2021)	*91*	*36*	*30*	*6*	*4.1*	*178 (II°)<br>143 (III°)*	*(178x30 + 143x 6) /36*	*172.1*	*3005*	*125*
Xiao<br>(2024)	*85<br>23*	*71.2<br>61.3*	*NR*	*NR*	*2.2<br>2.2*	*160<br>intermediate*	*NA<br>NA*	*160<br>160*	*4849<br>4250*	*202<br>177*
Zhu<br>(2021)	*109<br>82*	*53.1<br>55.2*	*NR*	*NR*	*2.59<br>2.29*	*160<br>Intermediate*	*NA*	*160<br>160*	*3635<br>3817*	*151<br>159*
Tan<br>(2021)	*149*	*68.9*	*33.7*	*35.2*	*2.5*	*178 (II°)<br>143 (III°)*	*(178x33.7 + 143x35.2)<br>/68.9*	*160.1*	*4739*	*197*
Bohem<br>(2019)	*38<br>38*	*50<br>49*	*NR<br>NR*	*NR<br>NR*	*5.2<br>4.7*	*160<br>intermediate*	*NA<br>NA*	*160<br>160*	*3859<br>3976*	*161<br>165*
Lin<br>(2018)	*80*	*44.9*	*NR*	*NR*	*4.44*	*160<br>intermediate*	*NA*	*160*	*3276*	*136*
Lindsey<br>(2020)	*156*	*31.7*	*NR*	*NR*	*3.9*	*160<br>Intermediate*	*NA*	*160*	*2471*	*103*
Nehemiah<br>(2018)	*97<br>24*	*30<br>46*	*NR<br>NR*	*NR<br>NR*	*4.2<br>3.5*	*160<br>Intermediate*	*NA<br>NA*	*160<br>160*	*2338<br>3391*	*97<br>141*
Nehemiah<br>(2016)	*203*	*31*	*NR*	*NR*	*4.0*	*160<br>Intermediate*	*NA*	*160*	*2381*	*99*
Rosenthal<br>(2018)	*36<br>60<br>40<br>9*	*44.5<br>36.5<br>39.6<br>38.1*	*NR<br>NR<br>NR<br>NR*	*NR<br>NR<br>NR<br>NR*	*5.4<br>4.3<br>3.8<br>3.3*	*160<br>Intermediate*	*NA<br>NA<br>NA<br>NA*	*160<br>160<br>160<br>160*	*3161<br>2901<br>3193<br>3409*	*132<br>121<br>133<br>142*
Kruse<br>(2025)	*90*	*36*	*NR*	*NR*	*6.5*	*160<br>intermediate*	*NA*	*160*	*2723*	*113*

Lamke base rates: 178 ml/m²/h for partial-thickness (II°), 143 ml/m²/h for full-thickness (III°), intermediate 160 ml/m²/h when depth distribution Not Reported (NR). Weighted calculation: (II°% x 178 + III°% x 143) / %TBSA$_{TOTAL}$; NA = not applicable; # Final TEWL : Liters/day: (ml/h x burned BSA x 24h)

Supplementary Table S5 – Detailed 24-hour input–output balance, estimated interstitial edema and TEWL/edema ratios in contemporary adult burn resuscitation cohorts.

Author	Year	N	Total TBSA (%)	ml/kg/%TBSA	Urine 24 h (ml)	Hourly I/O ratio	Calculated Edema (ml)	TEWL/Edema Ratio
Belba	2021	22	39	3.10	2282	0.1	4695	0.587
Meuli	2021	29	60	5.8	-	.	19111	0.231
		10	84	5.9	.	.	31338	0.150
Le Tacon	2023	230	38.6	4.3	1661	0.199	7637	0.349
Greenhalgh	2023	579	31.6	4.6	1879	0.220	6840	0.393
Chiao	2018	13	68	3.6	1636	0.133	9844	0.488
Tridente	2025	198	27.5	3.4	1312	0.219	3214	0.643
Lindhal	2023	46	32	5.9	1075	0.438	9779	0.246
Gardiner	2025	30	32	2.2	1339	0.146	1720	1.488
		14	42	3.5	861	0.394	9005	0.363
		80	33	5.9	2112	0.223	10211	0.247
Abdelmootal	2024	26	34.1	6.3	1783	0.309	12994	0.207
		26	33.5	6.1	1758	0.299	12223	0.209
Kahn	2025	213	31.8	3.3	2408	0.113	4084	0.563
Adibifar	2021	2822	30.8	5.1	1710	0.245	7795	0.288
Aigner	2025	892	23	3.0	-	-	1441	1.244
		444	40	3.7	-	-	6800	0.444
Comish	2021	91	36	4.1	461	0.750	9285	0.323
Xiao	2024	85	71.2	2.2	1296	0.114	5631	0.861
		23	61.3	2.2	1273	0.114	4137	1.027
Zhu	2021	109	53.1	2.6	1591	0.102	3909	0.929
		82	55.2	2.3	1632	0.086	2771	1.421
Tan	2021	149	68.9	2.5	1371	0.116	6031	0.786
Bohem	2019	38	50	5.2	2800	0.153	15634	0.246
		38	49		3800	0.106	14078	0.275
Lin	2018	80	44.9	4.7	1997	0.195	9910	0.330
Lindsey	2020	156	31.7	3.9	2722	0.121	5662	0.436
Nehemiah	2018	97	30	4.2	-	-	5747	0.406
		24	46	3.5	-	-	6754	0.502
Nehemiah	2016	203	31	4.05	-	-	5930	0.401
Rosenthal	2018	36	44.5	5.4	1278	0.278	10345	0.305
		60	36.5	4.3	1779	0.200	7836	0.370
		40	39.6	3.8	1718	0.227	10150	0.314
		9	38.1	3.3	1331	0.341	11837	0.287
Kruse	2025	90	36	6.5	1661	-	13218	0.206

Supplementary Table S6 – Historical datasets underpinning the edema regression model (fluid input, urine output, weight gain) and derivation of the interstitial edema equation.

Author	*Year*	*Weight (kg)*	*%TBSA*	*Input (ml)*	*Output (ml) (urine)*	*I-O (ml)*	*Weight Gain (%)*	*Gain Kg→ml*	*Estimated Edema(*)*
Du Ge Bing	*1991*	*80.8*<br>*80.2*<br>*82*	*43.5*<br>*49.8*<br>*47.4*	*10035*<br>*11657*<br>*18256*	*1435*<br>*1867*<br>*1673*	*8600*<br>*9790*<br>*16853*	*2.38*<br>*7.88*<br>*10.69*	*3347*<br>*4891*<br>*11190*	*3343*<br>*4908*<br>*11190*
O'Mara	*2005*	*88.5*<br>*87*	*50*<br>*52*	*22000*<br>*12300*	*1635*<br>*1585*	*20364*<br>*10713*	*18.05*<br>*6.13*	*14764*<br>*5505*	*14764*<br>*5505*
Tanaka	*2000*	*57*<br>*58*	*63*<br>*53*	*10773*<br>*16097*	*1505*<br>*1809*	*9268*<br>*15097*	*9.2*<br>*17.8*	*4047*<br>*9902*	*4047*<br>*9129*
Vachlou	*2010*	*85.6*<br>*70*	*23.5*<br>*32.5*	*8450*<br>*8650*	*2671*<br>*1932*	*5779*<br>*6718*	*2.92*<br>*2.0*	*1830*<br>*2021*	*1830*<br>*2021*

[Estimated Edema = 0,9154 x (fluid Input [ml] - Urine Output [ml]) - 4047.1]; R^2 0.907.

Supplementary File S1 – Evaporative Flux and Daily Water Loss: Fundamental Equations

1. ***Evaporative Power***

$$Ev\ (W/m^2) = h_e \cdot A \cdot \omega \cdot (P_{H2O(Skin)} - P_{H2O(Air)})$$

where $h_e \approx 2.2 \times (12.1 \times \sqrt{airspeed})$ g/(s·m²·°C) is the evaporative heat transfer coefficient.

2. ***Daily water loss per body surface***

$$Ev\ (ml/kg/day) = [Ev\ (W/m^2) \times BSA\ (m^2) \times 8.64 \times 10^4\ sec]/(weight\ (kg) \times 4186\ J/kcal \times 580\ kcal/L)$$

3. ***Air vapor pressure estimation***

$$P_{H2O(Air)}\ (mmHg) = \exp\ [18.6686 - (4030.183/\ (T\ °C + 235))] \times (RH/100)$$

Evaporative loss from burns: 580 kcal/L (1/3–2/3 BMR in 70 kg adults).

Key Determinants:

1. Vapor pressure gradient (wound 33–37°C vs. air).
2. Ambient T/RH (low RH/high T ↑ loss).
3. Airflow (0.6 m/s doubles loss).
4. Exposed area (0.6 × TBSA).
5. Wettedness ω (0–1): Fraction of surface with free water for evaporation; ≤0.25 in dry/thermoneutral wounds, ↑1 with exudation.

Supplementary File S2 – Sensitivity analysis

Scenario	*TEWL/Edema Ratio = 1 occurs at (ml/Kg/%TBSA)*	*R^2 power-law Regression (vs ml/Kg/%TBSA)*	*P value*	*TEWL/Edema Ratio = 1 occurs at (I/O Ratio)*	*R^2 hyperbolic Regression (vs I/O ratio)*	*P value*
Nominal Parameters (Lamke-derived TEWL, original edema regression)	***2.85***	***0.82***	***<0.0001***	***0.302***	***0.8634***	***<0.0001***
Lamke base-rate varied -30%	***2.00***	***0.87***	***<0.0001***	***0.211***	***0.912***	***<0.0001***
Lamke base-rate varied +30%	***3.71***	***0.71***	***<0.0001***	***0.393***	***0.721***	***<0.0001***
Edema Regression Intercept (-4047 ml) varied -50%	***2.65***	***0.85***	***<0.0001***	***0.285***	***0.899***	***<0.0001***
Edema Regression Intercept (-4047 ml) varied +50%	***3.05***	***0.78***	***<0.0001***	***0.332***	***0.812***	***<0.0001***
Edema Regression Intercept varied +/-20%	***2.95***	***0.80***	***<0.0001***	***0.318***	***0.835***	***<0.0001***
Ambient vapor pressure gradient varied -40% (wet)	***2.15***	***0.65***	***<0.0001***	***0.228***	***0.580***	***<0.0001***
Ambient vapor pressure gradient varied +40% (dry)	***3.85***	***0.75***	***<0.0001***	***0.421***	***0.620***	***<0.0001***
Worst Case simultaneous variation (all cases moved in the direction that most weakens the relationship	***1.89***	***0.70***	***<0.0001***	***0.199***	***0.721***	***<0.0001***
Best Case simultaneous variation (all cases moved in the direction that most strengthens the relationship)	***4.48***	***0.88***	***<0.0001***	***0.541***	***0.921***	***<0.0001***

Supplementary File S3 – Bedside worksheet

Rapid calculation of TEWL/edema ratio – Burn Unit Worksheet

Patient: ________________________ Weight: ______ kg BSA: ______ m² %TBSA burned: ______ %

1. Predominant depth → Lamke base rate:
   ☐ Full-thickness → 143 ml/m²/h
   ☐ Partial-thickness → 178 ml/m²/h
   ☐ Mixed/unknown → 160 ml/m²/h

2. Room T: ______ °C RH: ______ % → (optional correction factor: ______)

3. Expected 24-h TEWL = (%TBSA × base rate × 24) / 1000 = ______ litres

4. Fluid administered at __ h post-burn: ______ ml

5. Estimated oedema = 0.9154 × (fluid input – urine output) – 4047 ml = ______ ml

6. Current TEWL/edema ratio = expected TEWL (prorated) / estimated oedema = ______

Interpretation

- > 1.0 → check for inadequate perfusion
- 0.6 – 1.0 → on target
- < 0.6 → restrict crystalloids / consider colloids / check for compartment syndrome
